# Global Transcriptomic Profiling Reveals Conserved and Phage-specific Responses to Phage Infection in *Escherichia Coli*

**DOI:** 10.1007/s00248-025-02665-3

**Published:** 2025-12-11

**Authors:** Edgar González-Villalobos, Alicia Aranda, Ana C. M. de Almeida, José L. Balcázar

**Affiliations:** 1 Catalan Institute for Water Research (ICRA-CERCA), 17003 Girona, Spain; 2 Research Division, Department of Public Health, Faculty of Medicine UNAM, 04510 Mexico City, Mexico; 3 Independent Environmental Consultant, 01100 Viterbo, Italy

**Keywords:** Phages, Phage-host interactions, Stress response

## Abstract

Although phages shape bacterial evolution and physiology, the specificity of host transcriptomic responses to phage infection remains incompletely understood. Here, we performed global transcriptomic profiling of *Escherichia coli* exposed to two lytic phages, ΦX174 and T4, and the temperate phage λ, to explore both conserved and phage-specific host responses. All infections induced stress-related genes, including SOS and general stress pathways, along with repression of anabolic processes such as purine and amino acid biosynthesis, suggesting a metabolic shift to conserve resources. Notably, ΦX174 strongly activated the phage shock protein operon, while both ΦX174 and λ selectively induced *soxS*, a regulator of oxidative stress. Despite infecting the same host, each phage triggered distinct transcriptional signatures. These findings highlight the complexity of bacterial responses and the value of transcriptomics in decoding host-phage interactions, offering insights into resistance, survival, and co-evolution.

**Supplementary Information:**

The online version contains supplementary material available at 10.1007/s00248-025-02665-3.

Bacteriophages, or phages, are the most abundant and diverse biological entities on Earth, playing a key role in shaping microbial communities by infecting bacteria, facilitating horizontal gene transfer, and driving bacterial evolution through sustained selective pressure [1]. During infection, especially via lytic cycles, phages can activate bacterial defense mechanisms that help mitigate cellular damage and enhance survival.

In *Escherichia coli*, infection by the lytic phage P1*vir* induces oxidative stress and activates the transcription factor *soxS*, which regulates genes involved in detoxifying reactive oxygen species [2]. Although the *soxS*-mediated response is well characterized, broader transcriptional changes during lytic phage infection remain largely unexplored. Most studies have focused on a few stress markers or well-known systems such as the phage shock protein operon (*pspA* to *pspE*), which responds to membrane stress during filamentous phage infection [3]. In contrast, the potential of lytic phages to trigger other stress-response pathways, such as those related to envelope integrity, protein homeostasis, or DNA repair has not been thoroughly investigated.

To better elucidate the complex bacterial responses to lytic phage infection, we performed transcriptomic profiling of *E. coli* ATCC 700078 following exposure to two lytic phages, ΦX174 (ATCC 13706-B1) and T4 (ATCC 11303-B4), and the temperate phage λ (ATCC 23724-B2). This approach allowed us to uncover both conserved and phage-specific patterns of stress-related gene expression.

Briefly, cultures were grown in Luria–Bertani broth at 37 °C for 4 h to a density of 10^8^ CFU/mL, and then independently exposed in triplicate to each phage at a multiplicity of infection (MOI) of 1.0. Under a Poisson model of phage adsorption, the expected fraction of cells receiving at least one phage particle is 1 − *e*^−MOI^; thus, at MOI = 1, approximately 63% of cells are predicted to be infected [4, 5]. Exposure was maintained for 1 h to account for differences in adsorption dynamics and latent periods among the phages, thereby ensuring that the bacterial population was broadly challenged (doubling time under our conditions was ~ 25 min) and allowing measurable physiological effects to develop. This design was intended to capture a population-level transcriptomic snapshot of host responses to phage challenge, rather than early infection kinetics. A triplicate control without phage was also included. After exposure, bacterial cells were collected by centrifugation at 2,500 g for 2 min and subjected to RNA extraction using the RNeasy Plus Mini Kit (Qiagen, Hilden, Germany), according to the manufacturer’s instructions. Ribosomal RNA was depleted, and cDNA libraries were prepared using the Illumina Stranded Total RNA Prep with Ribo-Zero Plus kit (Illumina, San Diego, CA). Sequencing (2 × 150 bp) was performed on a NovaSeq 6000 platform (Illumina, San Diego, CA). Reads were trimmed, quality-checked, and initially aligned to the *E. coli* ATCC 700078 genome (= *E. coli* WG5; GenBank accession CP024090). To ensure robustness, reads were also aligned to *E. coli* K-12 substr. MG1655 [6] using Bowtie2 with default parameters (https://github.com/BenLangmead/bowtie2). This strain was used as the reference genome for RNA-seq analysis due to its extensive curation and functional annotation. This enabled reliable read mapping, differential expression assessment, and pathway interpretation. Differential expression and enrichment analyses were performed using the edgeR [7] and limma [8] packages from Bioconductor (https://bioconductor.org) in combination with the DAVID database [9].

Because the 1-h time point likely includes cells at different infection stages and potentially a minority of surviving or resistant cells, our analyses emphasize global transcriptional patterns at the population level. This population-snapshot interpretation is consistent with established RNA-seq studies of phage-host interactions [10, 11] and reflects the collective physiological state of the culture rather than stage-specific infection responses. Based on established infection kinetics, this 1-h snapshot is expected to capture early-to-mid infection for ΦX174 and T4, which initiate host takeover rapidly after adsorption, whereas λ-infected cells are likely in early infection or entering the lysogeny-lysis decision phase. Consequently, the transcriptomic profile reflects a mixed population in which cells occupy different infection stages depending on the phage. This clarification indicates that the observed responses represent aggregated population-level effects rather than synchronized infection kinetics. Such an approach enables the identification of overarching functional trends across treatments.

Consistent with this population-level perspective, gene ontology enrichment analysis revealed that while some enriched categories were represented by only a few genes, thus providing limited evidence of pathway-level activation, others such as flagellar assembly and chemotaxis were supported by multiple genes, indicating more robust and coordinated transcriptional changes. Reflecting this variability, genes associated with flagellar rotation, chemotaxis, fimbriae and flagellum biogenesis, phosphate transport, and bacterial stress responses, including genes involved in the SOS response, were upregulated following phage exposure (Fig. 1a). In contrast, downregulated genes were primarily associated with nitrogen fixation, purine biosynthesis, glycerol metabolism, and amino acid biosynthesis, including lysine and branched-chain amino acids (Fig. 1b). These transcriptional shifts suggest a reprogramming of metabolism away from anabolic activity, possibly to conserve energy or restrict resources available to the phage [12].

**Fig. 1:**
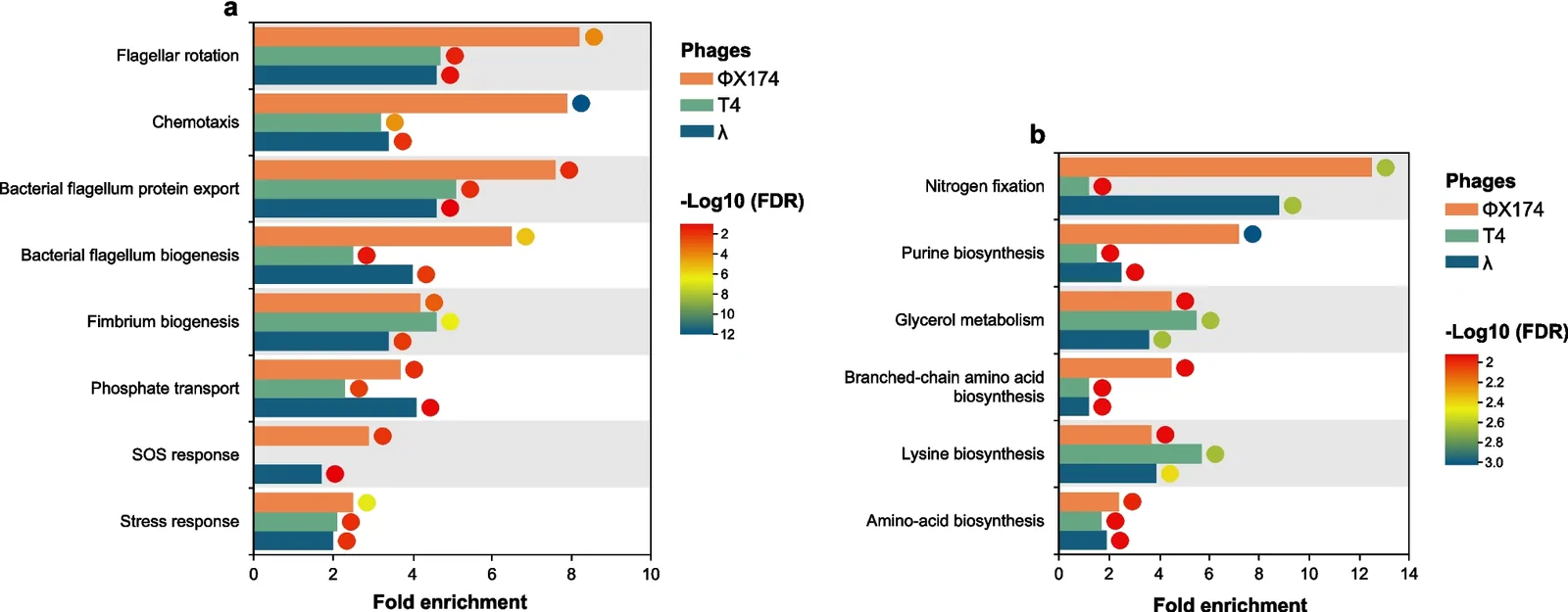
Gene ontology (GO) enrichment analysis following phage exposure. Fold enrichment (bars) and false discovery rate (FDR)-corrected *p*-values (circles) for significantly enriched GO biological processes among upregulated (a) and downregulated (b) transcriptomic responses to the lytic phages ΦX174 (orange) and T4 (green), and the temperate phage λ (aegean)

To further delineate these responses, we next analyzed individual differentially expressed genes. This assessment revealed both shared and phage-specific transcriptional responses (Supplementary Figure S1). A closer inspection showed consistent induction of *dinI*, *umuC*, *umuD*, *recA*, *sulA*, *yafP*, and *dinB*, with *recX* displaying the strongest upregulation among these genes (log_2_FC = 3.14, 3.58, 4.69 for λ, ΦX174, and T4, respectively) (Fig. 2). These genes are key markers of the bacterial SOS response, which is typically activated in response to DNA damage or replication stress [13]. Their coordinated upregulation suggests that phage infection, particularly with ΦX174, induces DNA- or replication-associated stress sufficient to trigger this pathway. Among them, *recA* and *recX* participate in homologous recombination and DNA repair, whereas *umuC* and *umuD* encode components of DNA polymerase V, which mediates mutagenic translesion synthesis. Moreover, *sulA* inhibits cell division to allow time for repair, while *dinI* and *yafP* have been associated with fine-tuning the SOS response and broader stress tolerance. Notably, *dinB*, which encodes DNA polymerase IV, was also significantly induced. This enzyme enables replication across damaged DNA templates, thereby contributing to stress tolerance but also potentially introducing mutations [14]. Although the SOS system is classically associated with genotoxic agents, it is well established that phage infection can also activate this pathway, particularly through mechanisms linked to replication stress, DNA damage or interference with host nucleases. In *E. coli*, some phages, including λ, are known to induce the SOS response during infection, and this regulatory connection has been previously documented [15–17]. Our results are therefore consistent with previous observations and expand them by providing transcriptome-wide evidence of SOS activation and by showing that the magnitude of induction varies among ΦX174, T4 and λ.

**Fig. 2:**
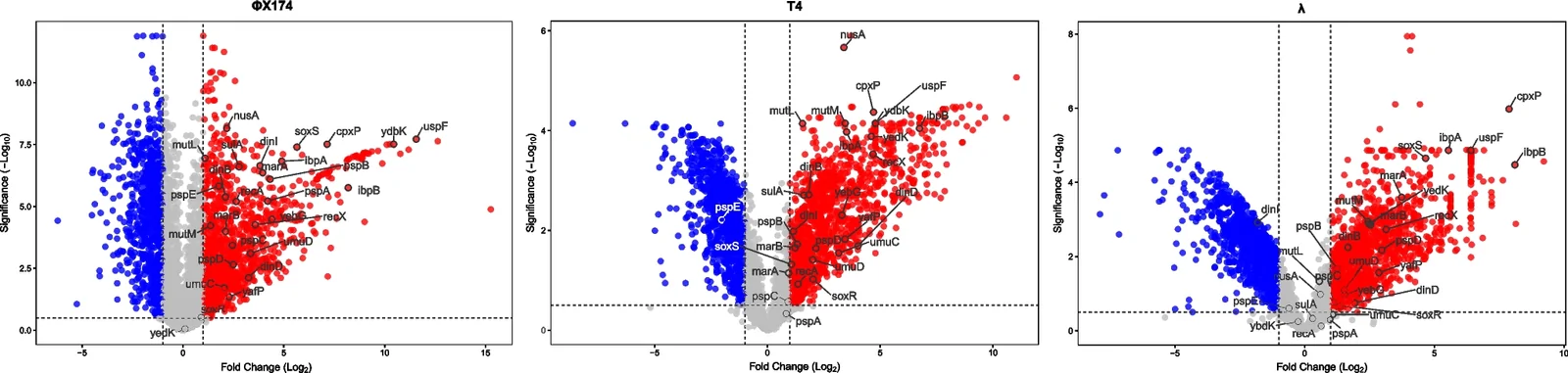
Phage exposure induces distinct transcriptomic profiles in *E. coli*. Volcano plots show the transcriptomic responses induced by the lytic phages ΦX174 and T4, and the temperate phage λ. Labeled genes represent the most strongly upregulated genes associated with the SOS response and general stress response, exhibiting the highest log2 fold changes

Several genes associated with general stress responses were also strongly induced across phage treatments, indicating that *E. coli* activates multiple protective pathways upon infection. Notably, the envelope-stress marker *cpxP* showed very high induction (log_2_FC = 7.88, 7.15, and 4.70 for λ, ΦX174 and T4, respectively), together with *uspF* (6.41, 11.57, and 4.79, respectively) and the heat-shock chaperone *ibpB* (8.11, 8.19, and 6.75, respectively), reflecting pronounced envelope and proteotoxic stress. Among these upregulated genes, *cpxP* encodes a periplasmic protein involved in the Cpx envelope-stress response, typically triggered by misfolded proteins or inner-membrane disruption [18]. The *uspF* gene, a member of the universal stress protein family, was also highly expressed, consistent with its role in adaptation to oxidative stress and nutrient limitation [19]. The *ydbK* gene, although less characterized, encodes a putative oxidoreductase likely involved in maintaining redox homeostasis. Increased expression of *nusA*, a transcription elongation and termination factor, may reflect host efforts to modulate transcription under phage-induced stress or interference by phage-encoded regulatory proteins [20]. In addition to its established role in transcription elongation, *nusA* is functionally linked to modulation of the SOS response and RNA polymerase activity under DNA damage [20], thereby reinforcing the connection between phage infection and DNA repair pathways. Furthermore, *ibpA* and *ibpB*, which encode small heat shock proteins functioning as molecular chaperones, were significantly upregulated, suggesting proteotoxic stress and the accumulation of misfolded proteins during infection.

We also observed selective induction of the phage shock protein (*psp*) operon, a stress-response system that helps maintain the proton motive force and protect inner-membrane integrity under envelope stress. This response was strongest upon ΦX174 infection, supporting the idea that small lytic phages disturb membrane-associated processes and thereby activate the Psp system. Notably, *pspA* and *pspB* reached their highest induction with ΦX174 (log_2_FC = 4.19 and 4.32, respectively), far exceeding the levels observed with λ or T4. In contrast, T4 and λ triggered only weak or partial activation. The coordinated upregulation of *pspA*, *pspB*, and *pspC* during ΦX174 infection suggests a targeted response to envelope stress, potentially acting early to limit membrane damage [21, 22].

In addition to envelope stress responses, we observed selective induction of *soxS*, a redox-sensitive regulator activated by oxidative stress. This occurred specifically upon ΦX174 and λ infection (log_2_FC = 5.65 and 4.66, respectively), but not with T4 (log_2_FC = 1.06), suggesting that these phages differentially trigger reactive oxygen species signaling. These findings are consistent with previous reports of strong *soxS* induction in *E. coli* following P1*vir* infection [2]. Our study extends these observations by demonstrating *soxS* activation at the transcriptome-wide level and by revealing distinct, phage-specific expression patterns. In line with this broader reprogramming, patterns linked to enhanced motility and chemotaxis may reflect a generalized stress adaptation rather than a phage-specific response. Such behavioral shifts could facilitate escape from localized phage predation or relocation to more favorable environments, consistent with previous descriptions of bacterial survival strategies under phage pressure [23, 24].

These phage-specific responses can also be interpreted in relation to the well-described lifestyles of ΦX174, T4, and λ. The strong activation of the *psp* operon by ΦX174 likely reflects its capacity, as a small icosahedral ssDNA phage whose lysis protein E targets MraY, to perturb envelope integrity [25]. The selective induction of *soxS* by ΦX174 and λ suggests oxidative stress linked to their infection mechanisms, with λ further contributing heterogeneity through its dual potential for lysogeny and lytic replication [15]. In contrast, T4, a large dsDNA lytic phage with a tightly regulated and rapid replication cycle, triggered comparatively weaker envelope- and oxidative-stress responses, consistent with its extensive host-takeover arsenal that rapidly reprograms bacterial physiology [26].

It should be noted, however, that infections were performed at an MOI of 1, which does not ensure that every cell was infected. It is therefore likely that uninfected cells contributed to the transcriptomic profiles, and the results should be interpreted as reflecting the population-level response of *E. coli* to phage exposure rather than the transcriptome of infected cells alone.

Taken together, phage infection in *E. coli* elicited a multifaceted stress response, including activation of the SOS system, envelope- and oxidative-stress pathways, and general adaptive mechanisms, along with repression of metabolic functions. This transcriptional landscape highlights substantial physiological changes even under controlled conditions. A key limitation of this study is the focus on a single post-exposure time point (1 h), which provides limited temporal resolution and does not capture early infection dynamics. Future studies should include multiple sampling points across the infection cycle to capture early and late dynamics. In summary, while all three phages triggered conserved stress programs (SOS, envelope, and general stress), each also produced distinct signatures consistent with its infection strategy. ΦX174 strongly activated the *psp* system, λ selectively induced oxidative-stress responses in line with its dual developmental program, and T4 elicited comparatively weaker host-stress signatures, likely due to efficient host takeover. This combination of shared and phage-specific patterns highlights the value of transcriptomics in disentangling conserved defense mechanisms from phage-specific adaptive responses.

## Supplementary Information

Below is the link to the electronic supplementary material.

Supplementary file1 (PDF 26 KB)

## Data Availability

Raw transcriptomic reads have been deposited in NCBI under BioProject accession no. PRJNA1287543.
