# Supplementary material for: Global Transcriptomic Profiling Reveals Conserved and Phage-specific Responses to Phage Infection in Escherichia Coli
Source: Microb Ecol. 2025 Dec 11;89:23. doi: 10.1007/s00248-025-02665-3 (PMC12795941; doi:10.1007/s00248-025-02665-3)
Supplement: Supplementary file 1 [file 248_2025_2665_MOESM1_ESM.pdf]

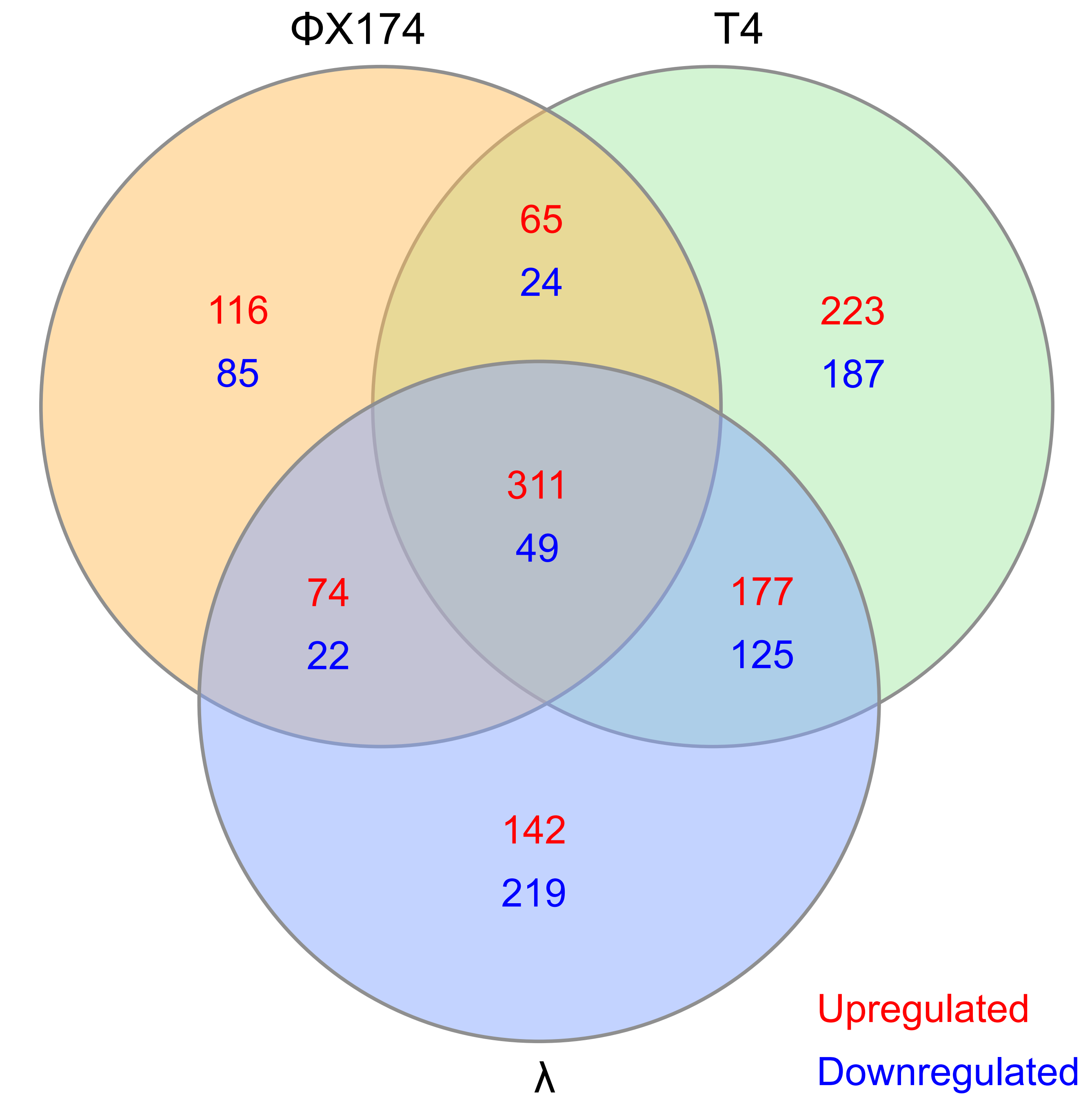
ΦX174
T4
116
85
65
24
223
187
311
49
74
22
177
125
142
219
λ
Upregulated
Downregulated
