# Supplementary figures and images for: Global Transcriptomic Profiling Reveals Conserved and Phage-specific Responses to Phage Infection in Escherichia Coli
Source: Microb Ecol. 2025 Dec 11;89(1):23. doi: 10.1007/s00248-025-02665-3 (PMC12795941; doi:10.1007/s00248-025-02665-3)

$\Phi$ X174

T4

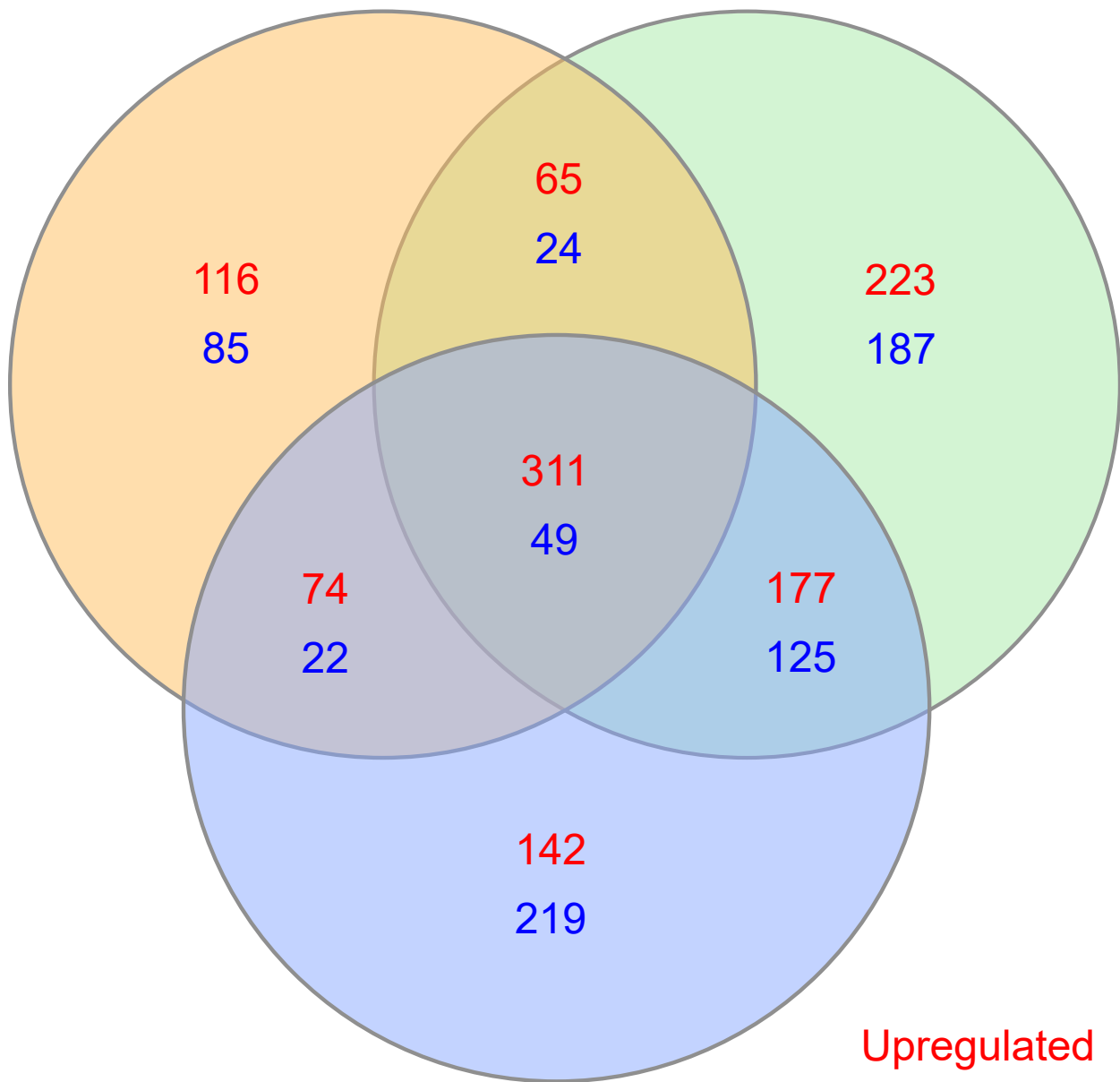

$\lambda$

Upregulated

Downregulated

Supplement: Supplementary file 1 — Supplementary file1 (PDF 26 KB) [file 248_2025_2665_MOESM1_ESM.pdf]
